# PD72 Supporting A Rapidly Expanding Program Of Work For A National Health Technology Assessment Agency: What Is The Impact?

**DOI:** 10.1017/S0266462325102031

**Published:** 2025-12-29

**Authors:** Louise Larkin, Michelle O’Shea, Debra Spillane, Marie Carrigan, Kieran Walsh, Máirín Ryan

## Abstract

**Introduction:**

The Health Information and Quality Authority (HIQA) Health Technology Assessment (HTA) Directorate, established in 2007, has rapidly expanded to more than 50 personnel working across seven teams. This expansion requires significant support for HTA activities, particularly in light of ongoing work to support implementation of the European Union Regulation on HTA. This abstract describes the impact of a recently established support function within the Directorate.

**Methods:**

The Strategy, Support, and Research (SSR) team was established in May 2023 to support HTA Directorate project delivery. The SSR team currently includes a Senior Management Team Lead, a Senior HTA Program Manager, a Librarian, and a Program Administrator. Specifically, the SSR team provides program management, administration, librarian support, corporate reporting, placement coordination, and risk management services, and supports research, learning and development, and quality improvement initiatives.

**Results:**

In 2024, the SSR team facilitated HTA activities by coordinating administration, project management, and librarian support for 16 projects, including 15 meetings with expert advisory groups comprising 285 key stakeholders. Twelve literature searches were conducted and 17 reports were published. Two Impact assessment reports were produced, which have 83 downloads and 3,217 social media impressions. The SSR team supported competitive grant funding applications securing more than EUR2.56 million in research funding, successfully applied for tenders with European HTA partners, and supported HIQA to successfully attain ISO 9001:2015 accreditation. This work supports outputs which inform health policy and decision-making in the healthcare ecosystem.

**Conclusions:**

With the increasing demand on HTA agencies, it is imperative that there is an effective support system in place to ensure that high quality outputs are delivered and stakeholders’ needs are met. The establishment of the SSR team in the HIQA HTA Directorate provides an example of how a dedicated team can provide support and impact for an expanding HTA agency.

